# Characterization of the Complete Chloroplast Genomes of *Buddleja colvilei* and *B. sessilifolia*: Implications for the Taxonomy of *Buddleja* L.

**DOI:** 10.3390/molecules23061248

**Published:** 2018-05-23

**Authors:** Jia Ge, Lei Cai, Gui-Qi Bi, Gao Chen, Weibang Sun

**Affiliations:** 1 Yunnan Key Laboratory for Integrative Conservation of Plant Species with Extremely Small Populations, Kunming 650201, China; gejia@mail.kib.ac.cn (J.G.); cailei@mai.kib.ac.cn (L.C.); 2 Key Laboratory for Plant Diversity and Biogeography of East Asia, Kunming Institute of Botany, Chinese Academy of Sciences, Kunming 650201, China; 3 University of Chinese Academy of Sciences, Beijing 100049, China; 4 Key Laboratory of Marine Genetics and Breeding (OUC), Ministry of Education, Qingdao 266100, China; fenghen360@126.com; 5 College of Marine Life Sciences, Ocean University of China, Qingdao 266100, China

**Keywords:** *Buddleja colvilei*, *Buddleja sessilifolia*, chloroplast genome, morphology, species delimitation, divergence times, threatened species, Himalayan alpine ornamental plant

## Abstract

*Buddleja colvilei* Hook.f. & Thomson (Scrophulariaceae) is a threatened alpine plant with a distribution throughout the Himalayas, also used as an ornamental plant. The name *Buddleja sessilifolia* B.S. Sun ex S.Y. Pao was assigned in 1983 to a plant distributed throughout the Gaoligong Mountains, but the name was later placed in synonymy with *B. colvilei* in the *Flora of China*. In this study we sequenced the complete chloroplast (cp) genomes of two individuals of *B. colvilei* and three individuals of *B. sessilifolia* from across the range. Both molecular and morphological analysis support the revision of *B. sessilifolia*. The phylogenetic analysis constructed with the whole cp genomes, the large single-copy regions (LSC), small single-copy regions (SSC), inverted repeat (IR) and the nuclear genes 18S/ITS1/5.8S/ITS2/28S all supported *B. sessilifolia* as a distinct species. Additionally, coalescence-based species delimitation methods (bGMYC, bPTP) using the whole chloroplast datasets also supported *B. sessilifolia* as a distinct species. The results suggest that the *B. sessilifolia* lineage was early diverging among the Asian *Buddleja* species. Overall gene contents were similar and gene arrangements were found to be highly conserved in the two species, however, fixed differences were found between the two species. A total of 474 single nucleotide polymorphisms (SNPs) were identified between the two species. The Principal Coordinate Analysis of the morphological characters resolved two groups and supported *B. sessilifolia* as a distinct species. Discrimination of *B. colvilei* and *B. sessilifolia* using morphological characters and the redescription of *B. sessilifolia* are detailed here.

## 1. Introduction

The Himalayan region is a center of diversity for the genus *Buddleja* L. (Scrophulariaceae) [1], harboring 75% of the Asian *Buddleja* species (18 of 24 species). Many species in the genus *Buddleja* are famous as ornamentals [2,3]. Indeed, *Buddleja colvilei* Hook.f. & Thomson, when discovered by Hooker in 1849, was described by him as “the handsomest of all Himalayan shrubs” [4,5]. *Buddleja colvilei* is a shrub or small tree [6,7], and as an ornamental plant, it was awarded the Royal Horticultural Society (RHS) First Class Certificate and the outstanding excellence for exhibition for the high ornamental value [4]. It is endemic to the eastern Himalayas, and has a distribution across altitudes of 1600–4200 m, in Nepal, India (Sikkim), Bhutan, and China (Tibet, Yunnan) [6]. In China, it is only found in Yadong, Tibet and the Gaoligong Mountains in Yunnan. The China Biodiversity Red List has evaluated *B. colvilei* as Vulnerable (VU) [8], although the species has not yet been assessed for the IUCN Red List.

**Figure 1:**
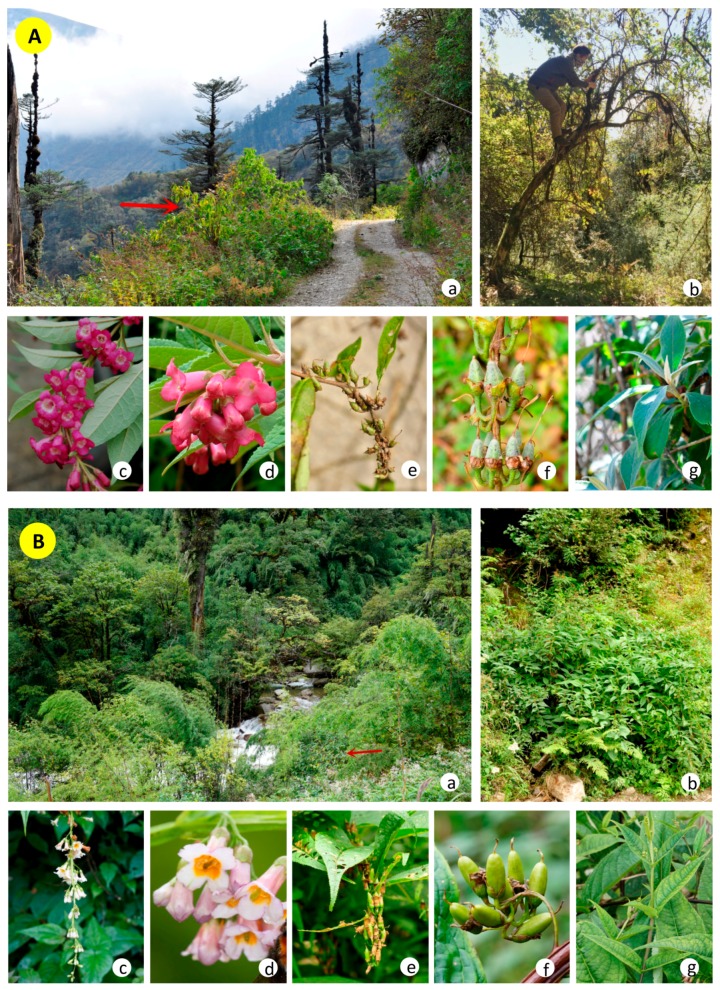
Morphological comparison of *Buddleja colvilei* and “*B. sessilifolia”*. (**A**) *Buddleja colvilei* plants from East Nepal and Tibet. (**B**) *Buddleja sessilifolia* plants from Gaoligong mountain. (**a**) habitat; (**b**) plant; (**c**) inflorescence; (**d**) flowers; (**e**) infructescence; (**f**) fruits; (**g**) leaves.

Species taxonomic designations are important to conservation, as species delimitations are the basis upon which conservation priorities are determined [9]. The taxonomy of this *Buddleja* is interesting, as our previous investigations found that *B. colvilei* plants from the western part of the range (East Nepal and Tibet) and the eastern part (Gaoligong Mountains) are distinct from each other in several ways: they are morphologically distinct from each other (Figure 1) in the sizes of the plants (small trees vs. subshrubs, Figure 1b); the color and size of the corolla (Figure 1c); the indumentum of the leaves, fruits and outside of the corolla (Figure 1d–g); the appearance and presence of the petiole on the leaves (Figure 1g); and the plants occupy different habitats (dry open areas and river banks (Figure 1a). The type specimen of *B. sessilifolia* was collected by T.T. Yu in 1938 [10] from the populations in the Gaoligong Mountains. In 1982 Li examined the same specimen and identified it as a new distribution record of *B. colvilei* in China [11]. However, ignoring the identification of Li, the *Flora Yunnanica* reported a new species based on this specimen in 1983, *Buddleja sessilifolia* B.S. Sun ex S.Y. Pao. After *B. sessilifolia* was published as a new species, the authors of the Flora of China (FOC) considered it morphologically identical with *B. colvilei* and published an article in 1988 sinking the name [12], and *B. sessilifolia* was synonymized with *B. colvilei* in FOC in 1992 [13]. These taxonomic designations were based on only a single specimen (the type of *B. sessilifolia*), which was in fruit, and had not been tested with molecular data. Moreover, although the fruits of these species are of a similar size, on closer examination we found that the fruits of “*B. sessilifolia*” are in fact glabrous, while the fruits of *B. colvilei* are covered with densely stellate tomentose hairs. Furthermore, because the species “*Buddleja sessilifolia*” published in the Flora Yunnanica was based on examination of the type specimen only, there are several mistakes or deficiencies in the description: the sizes of leaves and inflorescences were not accurate, the length and width of the corolla tube were not detailed (which are typically shorter than *B. colvilei*), the color of the corolla was not described, and the phenology description was not accurate.

Cytological research suggests that there are several ploidy levels occurring in this species, including 8*x*, 16*x* and in some instances extremely high ploidy levels of 24*x* [14,15]. The flowers and fruits of *B. colvilei* are larger than the typical butterfly bush (*B. davidii*) and other Buddlejas, which may be a result of the high ploidy. Polyploidy is thought to indirectly promote adaptation in alpine plants to higher elevations, enabling more rapid adaptation for niche shifts in the allopatric ranges [16,17,18]. The different morphological types from different habitats maybe a result of different ploidy levels adapted to the high altitude in the Himalaya, or to vicariance of different species during the uplift of the Himalayas. Thus, the taxonomy of *B. colvilei* is worth studying. Additionally, *B. colvilei* is an ideal species in which to conduct studies of alpine reproduction, adaptation to alpine environments, the study of speciation and evolution of *Buddleja*, and providing breeding stock for the development of new cultivars.

Owing to the lack of recombination, the low mutation rates, and uniparental inheritance on most occasions, the chloroplast genome is excellent for the study of plant speciation and evolution, especially when studying polyploids, as homeologous recombination, paralogy, and aneuploidy can make the use of nuclear genes problematic [19,20,21]. The chloroplast (cp) genome has been widely used for investigating phylogenetic relationships and discovering molecular markers for use in DNA barcoding to identify and discriminate between plant species, and in the discovering of new species [22,23,24].

To date, this study represents the first complete chloroplast genome study for species delimitation in *Buddleja*. Five individuals were collected from across the Himalayas, from five different populations, including three populations from the Gaoligong Mountains that were once recognized as *B. sessilifolia*. We sought to determine the complete chloroplast genome sequence of plants from each of these five populations, to clarify the taxonomic status of “*B. sessilifolia*” (three populations of the five) through phylogenetic analysis and the coalescence-based species delimitation methods (bGMYC, bPTP), to describe the structure of the complete chloroplast genome, and give a detailed taxonomic treatment of *B. sessilifolia* based on morphological and molecular characters. The results will provide taxonomic clarification of these *Buddleja* species and improve our understanding of *Buddleja* speciation and evolution.

## 2. Results and Discussion

### 2.1. Phylogenetic Analysis and Species Delimitation

**Figure 2:**
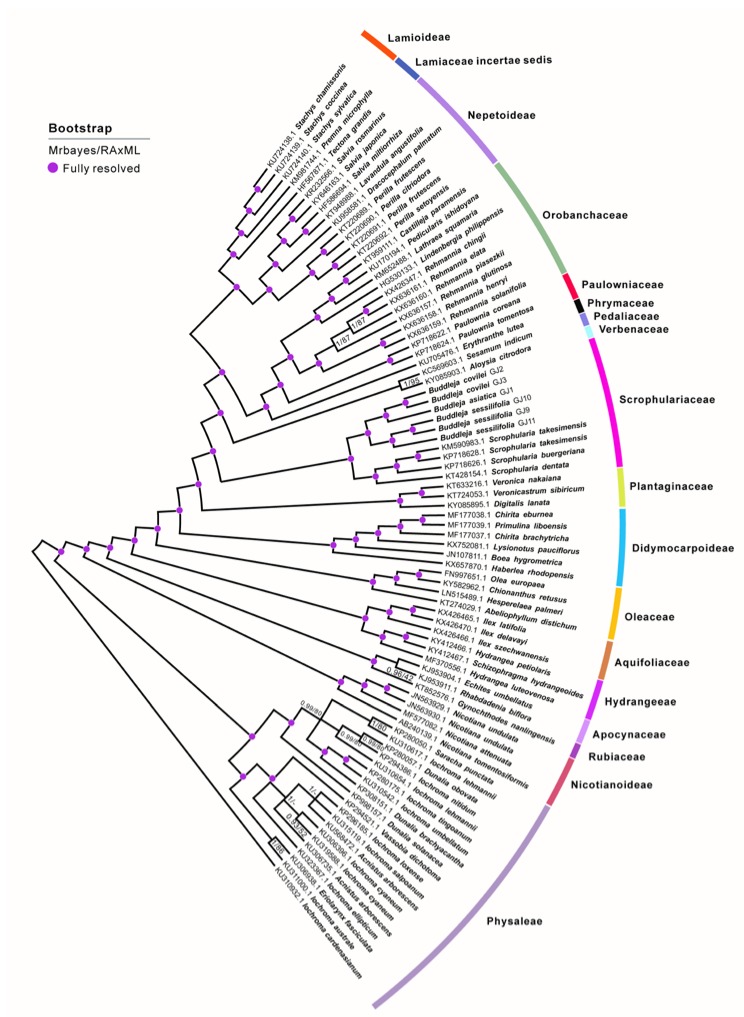
Molecular phylogenetic tree based on the complete cp genome of several representative species in the Scrophulariaceae family and related families and subfamilies. The tree was constructed using Bayesian and maximum likelihood (ML) analyses. Bootstrap values/Bayesian posterior probabilities (≥95%) are shown above branches. Purple dots mark nodes where both bootstrap values and Bayesian posterior probabilities are 100%.

Previous phylogenetic study suggests that *B. colvilei* forms a sister group to *B. asiatica* + *B. bhutanica*, and that these three species together form a sister group to the rest of Asian *Buddleja* species [25]. We therefore chose *B. asiatica* as reference to determine the taxonomic status of *B. sessilifolia*. To study the phylogenetic position of *B. colvilei* within the Scrophulariaceae family, whole cp genomes of *B. asiatica*, a further four cp genomes from three species of *Scrophularia* and 75 individuals of 72 species from related families and subfamilies were selected for analysis (Tables S1 and S2). Phylogenetic analysis revealed that all individuals from the Scrophulariaceae formed a monophyletic clade, as did the *Buddleja* species (Figure 2).

**Figure 3:**
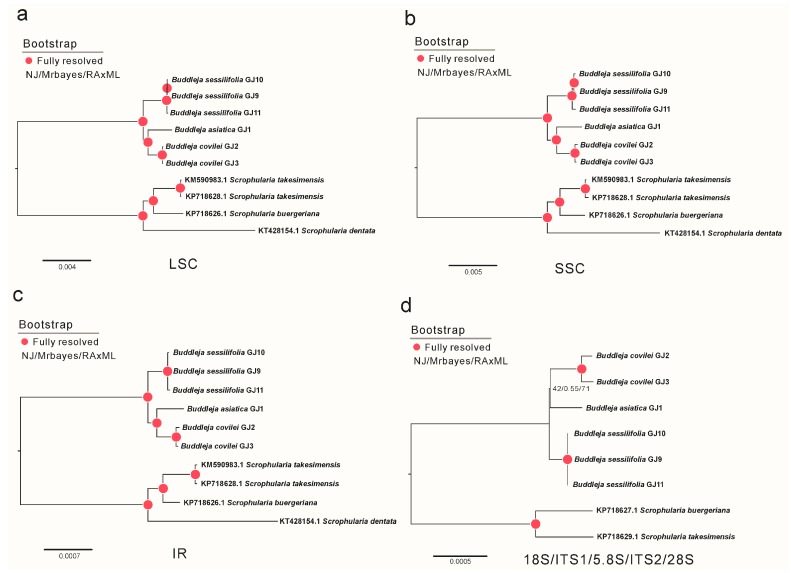
Molecular phylogenetic tree based on four partitioned datasets of the Scrophulariaceae family. (**a**) Phylogenetic tree built using LSC; (**b**) Phylogenetic tree built using SSC; (**c**) Phylogenetic tree built using IR; (**d**) Phylogenetic tree built using 18S/ITS1/5.8S/ITS2/28S. Trees were inferred using neighbor-joining (NJ), Bayesian and maximum likelihood (ML) analyses respectively. Bootstrap values of NJ/ML and Bayesian posterior probabilities (≥95%) are shown above branches. Red dots indicated nodes where bootstrap values of NJ, MLand Bayesian posterior probabilities are all 100%.

The analysis strongly supported *B. sessilifolia* as a distinct species, with the individuals from the three *Buddleja sessilifolia* populations resolved in a clade that was sister to *Buddleja asiatica* + *B. colvilei* (100% bootstrap support), rather than with *B. colvilei*. Moreover, the individuals from all the *B. colvilei* populations resolved as sisters to *B. asiatica*, with a bootstrap value of 100%. The evolutionary relationships resolved in this study are consistent with previous research [25,26,27] but not with previous taxonomic descriptions [6,11,12,13]. We used the large single-copy region (LSC), small single-copy region (SSC), inverted repeat (IR) and the nuclear genes 18S/ITS1/5.8S/ITS2/28S separately to study the phylogenetic position of *B. colvilei* and *B. sessilifolia* with respect to *B. asiatica* and three *Scrophularia* species. All the phylogenetic data from our study showed that the three individuals of *B. sessilifolia* resolved in a clade that was a sister group to the clade of *B. colvilei* + *B. asiatica,* and their phylogenetic positions remained stable. The highly conserved chloroplast genome structures therefore indicated that *B. sessilifolia* and *B. colvilei* are distinct species (Figure 3).

**Figure 4:**
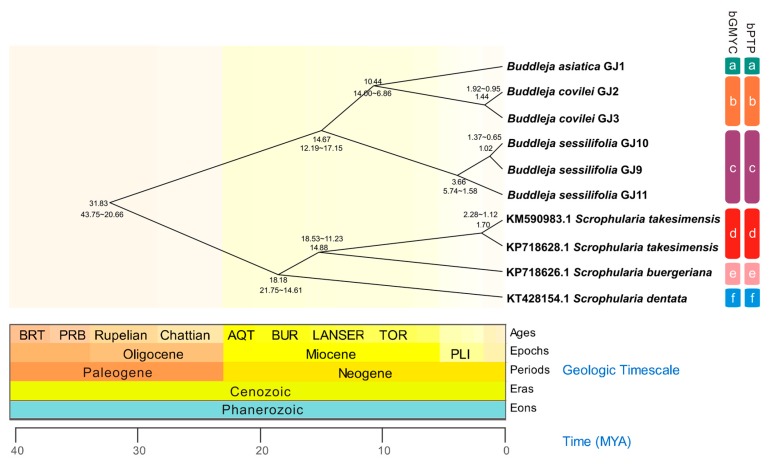
BEAST chronogram of the Scrophulariaceae family based on the complete cp genomes. Columns on the right represent the results of species delimitation from two different tests. Geologic timescale was obtained from TIMETREE, time is shown in millions of years.

The coalescence-based species delimitation methods (bGMYC, bPTP) implemented with the whole chloroplast datasets resolved six species (a–f). They were confirmed as segregated entities with the probability threshold >0.5, and the *B. sessilifolia* was also supported as a distinct species with a divergence time around 14.67 Ma (95% Highest Posterior Density, HPD: 12.19–17.15 Ma), earlier than those of *B. asiatica* and *B. colvilei* (Figure 4). Our results indicated that the *B. sessilifolia* lineage is early diverging among the rest of the Asian *Buddleja* species, with a divergence time earlier than those of *B. colvilei* and *B. asiatica*, which were previously considered as the basal groups of Asian *Buddleja* [25]. The divergence of the three *Buddleja* species may be related to the vicariance during the uplift of the Himalayas during the orogeny in the Miocene [28]. Furthermore, the divergent of populations of *B. sessilifolia* may be related to the uplift of the Gaoligong Mountains, which occurred during or after the Late Pliocene [29].

The conservation status of *Buddleja colvilei* has been assessed as VU [8], and based on the results of our study and information from herbarium specimens, including specimens from the Kunming Institute of Botany, CAS (KUN) and Chinese Virtual Herbarium (http://www.cvh.ac.cn) *B. sessilifolia* should be regarded as endangered. As most of the range of both *B. colvilei* and *B. sessilifolia* is located at or near the boundaries of several countries, trans-boundary conservation of these two species should be conducted in the future. Furthermore, the mechanism by which *B. colvilei* became polyploid, its adaption to the alpine environments, and observed pollinator shifts from birds to bumblebees in *Buddleja* are all worth studying in greater detail.

### 2.2. Genome Organization, Features and the Comparisons between B. colvilei and B. sessilifolia cp Genomes

**Figure 5:**
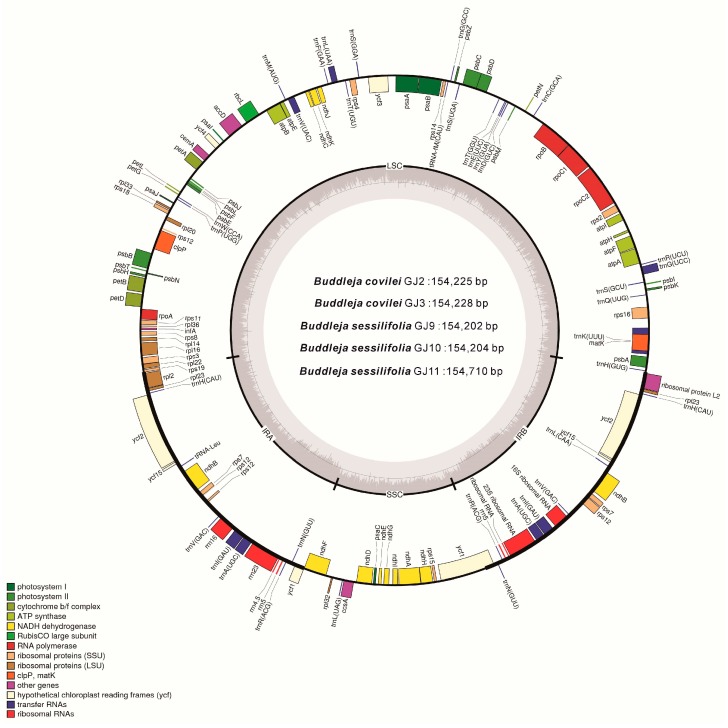
Gene map of *Buddleja colvilei* and *B. sessilifolia* with *Buddleja colvilei* (GJ2) as reference. Genes drawn inside the circle are transcribed clockwise, and outside of the outer layer circle are transcribed counterclockwise. The colored bars indicate known protein-coding genes, tRNA, and rRNA. The dashed darker gray area in the inner circle denotes GC content, and the lighter gray area indicates AT content. LSC (Large Single Copy) region; SSC (Small-Single Copy) region, and IR (Inverted Repeat).

The complete chloroplast genomes of both individuals of *B. colvilei* and the three individuals of *B. sessilifolia* were found to have a total length ranging from 154,202 bp to 154,710 bp. Both *B. colvilei* and *B. sessilifolia* had a quadripartite structure like most land plants, consisting of two inverted repeats (IRs) separating the LSC region and the SSC region (Table 1 and Figure 5). In total, the LSC regions accounted for 85,179 to 85,687 bp (55.24 to 55.39%), SSC regions accounted for 17,905 to 17,920 bp (11.57 to 11.62%) and IR regions accounted for 25,553 to 25,559 bp (16.52 to 16.57%). Total GC content was found to be from 38.07 to 38.11%. Like most plants [30,31], the GC content is unevenly distributed throughout the cp genomes of the *Buddleja* genus. In *B. colvilei*, the IRs had the highest GC content (43.23%), the LSC region had 36.19% and the SSC region had 32.20% GC content. Similar results were found in *B. sessilifolia* (Table 1). The previous studies indicated that the high GC content in the IR regions is likely to have been caused by the high percentages of GC nucleotides found in the four rRNA genes *rrn4*.*5*, *rrn5*, *rrn16*, and *rrn23* [31,32]. This phenomenon is very commonly found in other plants [24,31,32,33].

The chloroplast genomes of *B. colvilei* and *B. sessilifolia* contained a total of 135 genes, with a 136th gene in GJ11 identified as a relocated segment of *rpl2.* All unique genes were arranged in the same order in both species, eighteen of which are duplicated in the IR regions. Among these unique genes, there were 80 protein-coding genes, 37 transfer genes and four rRNA genes (Table 2). Thirteen genes contained a single intron (comprising eight protein-coding and five tRNA genes) and three encoded two introns (*ycf3*, *clpP* and *rps12*). The *ycf1* gene was found to be located between the IRa and the SSC regions. The *ycf1* gene had premature stop codons in the coding sequence, and has been annotated as a pseudogene here and in other angiosperm chloroplast genomes [34,35]. In addition, the pseudogene *ycf*15 was also identified.

**Figure 6:**
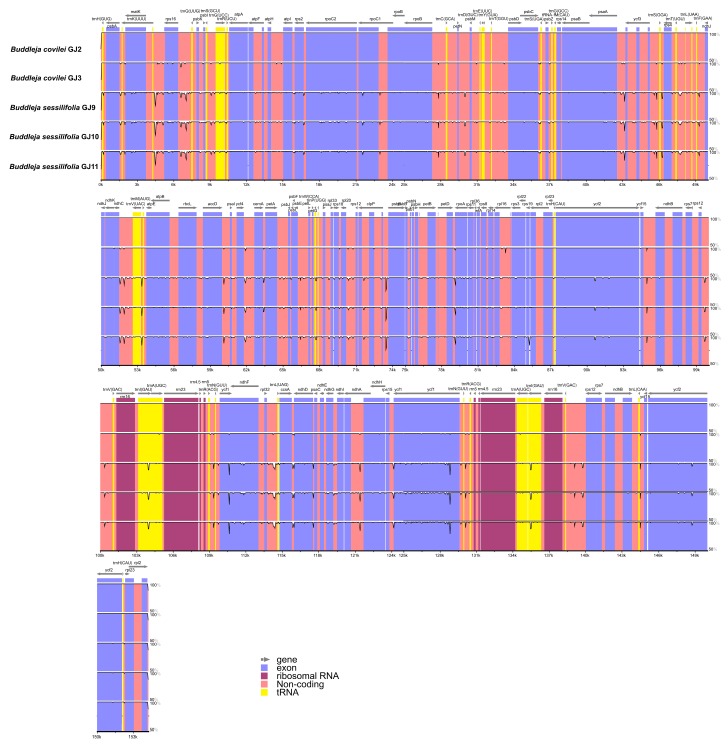
Sequence alignment of chloroplast genomes from five individuals of two *Buddleja* species, *Buddleja colvilei* (GJ2) used as reference, by using a 50% identity cutoff. Gray arrows show direction and position of each gene. The colored areas indicate exons, rRNA, Non-coding regions, and tRNA. The Y-axis represents the percentage identity between 50–100%.

The overall sequence variation in the cp genomes of the five individuals was plotted using the mVISTA program. The results suggest that the organization of the cp genomes of the two *Buddleja* species is highly conserved (Figure 6). However, certain dissimilarities were identified between species, occurring more frequently in the noncoding regions than the coding regions. Most divergence was found between species, with GJ 2 and GJ 3 (the *B. colvilei* individuals) being more similar to each other than to GJ 9, GJ 10 and GJ 11 (the *B. sessilifolia* individuals).

**Figure 7:**
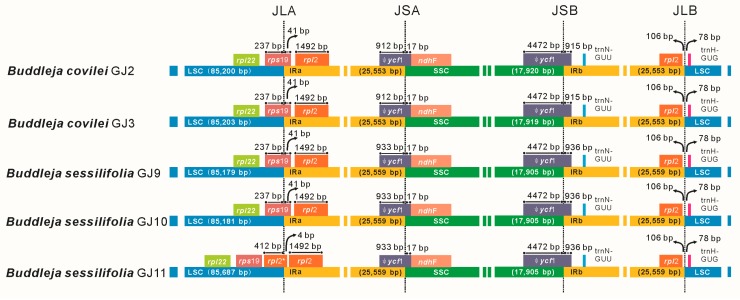
Comparison of the borders of the LSC, SSC, and IR regions from the cp genomes of five individuals from two *Buddleja* species. * Indicates a gene segment. Ψ Indicates a pseudogene. The figure is not drawn to scale.

In the cp genome, the IR/LSC boundaries are not static, but are subject to dynamic and random processes that allow expansions and contractions [32], which are important evolutionary events and have measurable influence on the size of the chloroplast genomes [36]. The IR junctions of most plastid genomes have been similarly described using the map points JLB (IRb /LSC), JSB (IRb/SSC), JSA (SSC/IRa) and JLA (IRa/LSC) [37]. A detailed comparison of the junctions of the IR/SSC boundaries within the five *Buddleja* plastid genomes is presented in Figure 7. The length of the IR regions was consistent within species, and in *B. sessilifolia* this region was 5 bp longer than in *B. colvilei*. The IRb/SSC boundary was located between the *ycf*1 pseudogene and *trnN*-GUU in all the sampled cp genomes studied here. The gene *trnN*-GUU is found at the IR/SSC boundary in most species, and it is considered as the ancestral IR/SSC endpoint [38]. There was expansion of the IR regions in the sample GJ11 into the extra *rpl*2 gene segment and *rpl*2 at the IRa/LSC, while in other individuals the IR regions expanded into the *rps*19 and *rpl*2 at the IRa/LSC. The relocation of an extra segment of *rpl*2 at the IRa/LSC boundary of GJ11, made the cp genome in this individual approximately 500 bp longer than those of other individuals. Previous studies have reported that the expansion of IR ranges from several bp to several kb in plants [38,39], results from the relocations of single genes or multiple genes [38,39,40,41] which are assumed to be an important mechanism of molecular evolution in chloroplasts [39,41]. The expansion and contraction of *rpl*2 has been detected numerous in land plants [38,42,43,44,45]. The *rpl*2 gene is located in the IR at the IR/LSC boundary and present in two copies in most plants [43]. It was confirmed that precise excision of *rpl*2 genes were resulted from the lack of intron, which has led to a model of loss that invokes RNA-mediated recombination as a causation mechanism [43,44]. However, the mechanism of the expansion of *rpl*2 remains unclear. In our analyses (whole cp genomes, three Cp data partitions (SSC, LSC and IR), and the nuclear genes 18S/ITS1/5.8S/ITS2/28S), the expansion at the IRa/LSC boundary of GJ11 did not lead to any change in the phylogenetic position of GJ11. This is probably because the sequence of the remaining cp genomes of the three *B. sessilifolia* individuals is highly conserved. The pseudogene *ycf*1 was completely contained within the IR region, and both JSB and JSA junctions share synapomorphic structural features; a *ycf*1 pseudogene of 4,472 bp size spans JSB, and a truncated *ycf*1 pseudogene of 912 or 933 bp spans JSA.

**Figure 8:**
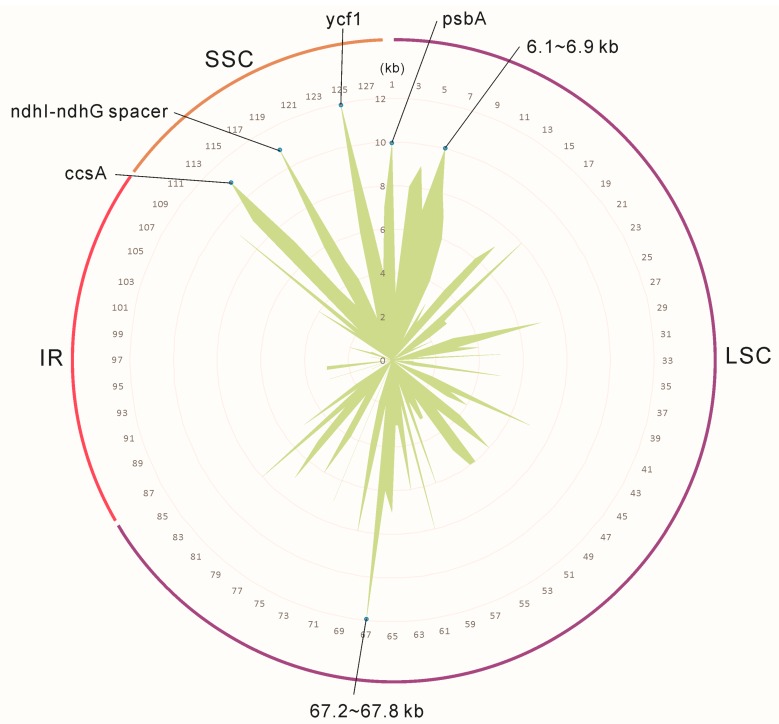
Distribution of the single nucleotide polymorphisms (SNPs) detected between *Buddleja colvilei* and *B. sessilifolia*.

A total of 474 single nucleotide polymorphisms (SNPs) were identified in our data as being informative in distinguishing between *B. colvilei* and *B. sessilifolia*, suggesting that *B. sessilifolia* should be regarded as a distinct species from *B. colvilei*. Most SNPs detected were in the LSC region, accounting for 71.73% of the total SNPs, and of these, 129 SNPs were located in coding regions, and 211 were located in non-coding regions. A further 23.84% were located in the SSC region, with 41 SNPs occurring in coding regions, and 72 in non-coding regions. The IR regions were more conserved than other regions, containing the fewest SNPs (4.43%), with nine occurring in coding regions and 12 in non-coding regions (Figure 8). Similar results from other land plants have been obtained by other studies [45], and there is evidence that the conserved or reduced substitution rate in the IR is due to the copy-dependent repair mechanism of the duplication [46]. Moreover, we found that, of all the genes, the *ycf*1 gene contains the most SNPs, indicating high divergence in this gene. The *ccs*A gene and the protein-coding gene *psb*A also contain more than ten SNPs each. The regions from 67.2 to 67.8 kb, 6.1 to 6.9 kb, and the *ndh*I-*ndh*G intergenic spacer region also contain numerous SNPs (Figure 8). These genes and regions could be used as potential barcoding sites for the further identification of and discrimination between *Buddleja* species.

### 2.3. Morphological Study and Taxonomic Treatment

A previous phylogenetic study indicated that *B. asiatica* and *B. bhutanica* were the most closely related species to *B. colvilei* [25]. In contrast to the large flowers of *B. colvilei*, in which the corolla tube is normally about 20 mm, *B. asiatica* bears almost the shortest flowers of all the Asian *Buddleja* species, with a corolla tube of 3–6 mm. Furthermore, the length of the capsule of *B. colvilei* is triple that of the 3–5 mm long capsules from *B. asiatica* [11]. Compared to other Asian *Buddleja* species, *B. asiatica* has narrower leaves, narrower inflorescences and narrower inflructescences (Figure S1), and is thus easy to identify. *Buddleja bhutanica* is very similar to *B. asiatica* but distinguished by its connate-perfoliate leaves [7]. As *B. asiatica* and *B. bhutanica* are easy to distinguish from *B. colvilei* and *B. sessilifolia*, in this study we concentrated on the morphological differences between *B. colvilei* and *B. sessilifolia*, and our taxonomic treatment is focused on these two species.

**Figure 9:**
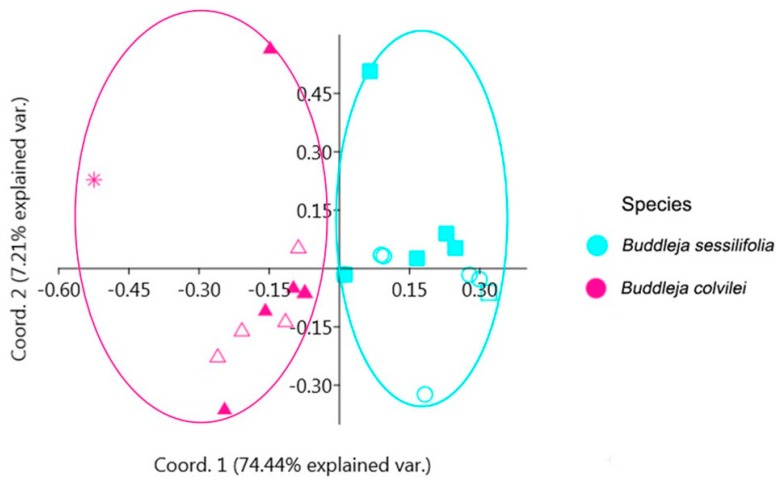
Principal Coordinate Analysis using six morphological characters measured in two species. The symbols represent the four populations: filled squares (K), rings (DZ), empty triangles (YD), filled triangles (GO), and star (Specimens).

We used five morphological characters (length of styles, length and width of fruits, and hairs on both ovary and capsules) measured from plants from two populations of each species, with the sixth morphological character length of corolla tube measured from specimens of *B. colvilei* and two populations of *B. sessilifolia* to do the Principal Coordinate Analysis (PCoA). GO and YD populations represent *B. colvilei*, and DZ and K populations represent *B. sessilifolia*. The results show two groups, supporting *B. sessilifolia* as a distinct species (Figure 9). The taxonomic treatment and comparison of the two species are given below.

***Buddleja sessilifolia*** B.S. Sun ex S.Y. Pao in Fl. Yunnanica 3: 5, Pl. 134, 1–4. 1983. —

in Fl. *Reipublicae Popularis Sinicae* 61, pl. 282–283. 1992. (synonymous with *B. colvilei*) —

in *Flora of China* 15, pl. 332. 

**Type:** China. Yunnan: Nujiang Prefecture, Gongshan County, Dulong River, in fruit,

3 Nov. 1938 *T.T. Yu 20946* (type: PE!)

**Re-description:**

Subshrubs, about 1 m tall. Branchlets quadrangular, with narrow wings, smooth glabrous. Leaf blade ovate oblong, 7.5–30 × 4.5–12.5 cm, smooth and glabrous on both sides, apex acuminate, base rounded, margin with slender teeth. Inflorescences terminal or axillary, symphitic cymes, 3–12(–61) cm long, covered with scattered stellate hairs. Calyx campanulate, 3–5.5 mm, tube 2–4.5 mm, lobes triangular, very short, ca. 1 mm long, smooth and glabrous on both sides; Corolla white or pink to pink-purple, yellow inside, 7.5–14 mm long; glabrous outside, pilose inside above the middle, especially at the throat, tube broadly cylindrical, 6–12 mm long, 3.5–7 mm in diam.; lobes suborbicular, 3–6.5 × 3–6.5 mm. Stamens inserted ca. 2 mm below mouth; anthers oblong, ca. 1 mm long, apex obtuse to apiculate. Ovary obovate, ca. 5 mm long, glabrous. Style 4–5 mm long, glabrous; stigma rod-shaped. Capsules oblong, 0.7–1.5 × 0.3–0.6 cm, glabrous, with persistent style. Seeds oblong, 1.5–2.0 mm, short-winged.

**Phenology:** —Flowering from June to August, fruiting from September to October.

**Taxonomic affinities:** —*Buddleja sessilifolia* can be easily differentiated from *B. colvilei* as they are procumbent subshrubs normally below two meters in size, with the quadrangular glabrous branches, the absence of a petiole on the leaves, the white to pinkish-purple corolla that is yellow inside and less than 2 cm in length, as well as the glabrous calyx, ovary and capsules. Comparison of the characteristics of these closely related species is detailed in Table 3.

The inside of the corolla of *B. sessilifolia* is yellow, which has been hypothesized to be a nectar guide. Interestingly, unpublished data shows that the main pigments of the floral nectar guide of *B. sessilifolia* contained several diterpenes (non-cyclic crocetin gentiobiose esters), instead of flavonoids [47]. These terpenoids have a conjugated double bond which can reflect UV light, whereas flavonoids can absorb UV light. Bees, flies, and butterflies are known to be very sensitive to UV light [48,49], and we suggest that the yellow nectar guide of *B. sessilifolia* may be a significant attractant for bumblebee pollinators. However, the purple to wine red floral color without nectar guides in *B. colvilei* could attract bird pollinators, a hypothesis supported by oral reports from Nepali residents: Kanxi and Tamang, from Goruwale, Mechi, Nepal.

## 3. Materials and Methods

### 3.1. DNA Extraction and Sequencing

The *B. colvilei* (GJ2, GJ3) and *B. sessilifolia* (GJ9, GJ10, GJ11) plant samples were collected from Goruwale, Mechi, Nepal and Yadong, Tibet, China, *B. sessilifolia* plant samples were collected from the Gaoligong mountain, Yunnan, China, and the Gaoligong Mountain, Myanmar. Table S2 gives details of the collections. Total genomic DNA was extracted from 100 mg fresh leaves using a modified CTAB method [50]. The complete cp genome sequencing was performed on the Illumina MiSeq 2000 (Illumina Inc, San Diego, CA, USA), at the Laboratory of Molecular Biology of Germplasm Bank of Wild Species in Southwest China, following the method of Yang [51]. The Illumina raw sequence reads were edited using the NGS QC Tool Kit v2.3.3 [52], with a cut-off value of 80 and 30 respectively for percentage of read length and PHRED quality score respectively. High-quality reads were assembled into contigs using the *de novo* assembler SPAdes 3.9.0 [53], using a *k*-mer set of 93, 105, 117, 121. The *de novo* contigs were assembled into complete chloroplast genomes by further connection using NOVOPlasty version 2.6.2 [54].

### 3.2. Phylogenetic Analysis and Species Delimitation

#### 3.2.1. Complete cp Genome Based Phylogenetic Analysis 

Total cp genomes from 85 individuals (Tables S1 and S2) of 78 related higher plants species and *Buddleja asiatica (*GJ1) were included in our analyses to resolve topology within the Scrophulariaceae (data not shown). The complete genomes reported for other species were downloaded from the NCBI GenBank database (Table S1) and were aligned using HomBlocks [55] with the Gblocks method [56], resulting in 47,802 aligned characters. Phylogenetic analyses were carried out using NJ (MEGA6 [57]), Bayesian (MrBayes v3.2.5 [58]) and maximum-likelihood (RA × ML version 8.1.12 [59]) methods. Ten independent ML searches were conducted, and the branch support was determined by computing 1000 non-parametric bootstrap replicates.

#### 3.2.2. Cp Data Partition 

The cp genomes were divided into the large single copy (LSC) regions and small single copy (SSC) regions, and the invert repeat regions (IR). We used these partitioned data sets in our neighbor-joining (NJ), Bayesian and maximum-likelihood (ML) analyses respectively for the ten Scrophulariaceae cp genomes. Both maximum likelihood and Bayesian analyses were used to verify the topology indicated in the tree derived from 85 whole cp genomes. These three-part alignments were constructed in MAFFT v7 [60] and trimmed using trimAl 1.2rev59 [61]. The length of aligned LSC, SSC and IR regions were 81,378 bp, 17,160 bp and 25,361 bp, respectively.

#### 3.2.3. 18S-ITS1-5.8S-ITS2-28S 

The 18S-ITS1-5.8S-ITS2-28S operons of the *Buddleja* species were recovered by homologous Blast searches using the counterpart operon of *Scrophularia takesimensis* (KP718629.1) and *S. buergeriana* (KP718627.1) against the raw DNA assembly results. HomBlocks [56] was also employed for gene alignment and concatenation (6254 characters). 18S-ITS1-5.8S-ITS2-28S operons were used to reconstruct the phylogeny by using three approaches: neighbor-joining (NJ), maximum likelihood, and Bayesian analyses.

#### 3.2.4. Model Selection 

The best substitution model for each dataset was determined by using jModeltest 2 [62] with Akaike information criterion (AIC). The Bayesian information criterion (BIC) indicated that GTR+G, TVM+G, TVM+G, TPM1uf+G and TrN+G models were the most appropriate for the whole cp genome alignments, LSC alignments, SSC alignments, IR alignments and 18S-ITS1-5.8S-ITS2-28S alignments, respectively. Maximum Likelihood (ML) and Bayesian phylogenetic trees were inferred with RA × ML version 8.1.12 [59] and MrBayes v3.2.5. [58] using the appropriate models.

#### 3.2.5. Maximum Likelihood 

ML analyses were performed with RA × ML version 8.1.12 [59] coupled with raxml-ng (https://github.com/amkozlov/raxml-ng) for its application of other substitution model, because RA × ML version 8.1.12 only uses the invariable (general time reversible) GTR model. The ML trees were inferred with the combined rapid bootstrap (1000 replicates).

#### 3.2.6. Bayesian Analyses 

Bayesian inference analyses (BI) were performed in MrBayes v3.2.5 [58]. The following settings were applied: 10,000,000 number of Markov chain Monte Carlo (MCMC) generations, including four chains each (three heated, heat parameter = default) with a sampling frequency of 1000 generations, each chain starting with a random tree. The first 25% of trees from all runs were discarded as burn-in and the remaining trees were used to construct a majority-rule consensus tree. The robustness of the resultant BI tree was evaluated using bootstrap probabilities. In the partitioned dataset and 18S-ITS1-5.8S-ITS2-28S operon data, genus *Scrophularia* was set as the outgroup.

#### 3.2.7. NJ Method 

For the NJ analyses, all trees were built under Maximum Composite Likelihood method with Gamma distribution rates (parameter = 4) using MEGA6 [57]. Robustness of nodes was assessed with 1000 NJ-bootstrap replicates using the Maximum Composite Likelihood method under Gamma Parameter 4. Trees were visualized in Figtree v1.4.2 [63].

#### 3.2.8. Species Delimitation 

Species boundaries in Scrophulariaceae were determined following Hassanpour’s methods [64] using the coalescence-based species delimitation methods implemented in bGMYC [65] and bPTP [66]. For the bGMYC analysis, 10,000 random ultrametric trees of the whole chloroplast dataset obtained from the posterior distribution of the BEAST analysis outputs were sampled as an input to integrate over the uncertainty of tree topology. BEAST v2.2.0 [67] was used to generate the chronogram tree, assuming a Bayesian relaxed-clock model. Molecular rates were allowed to vary around an average value among lineages, by imposing an uncorrelated lognormal clock of evolutionary rates. The Yule process prior was applied in this analysis. Fossil-derived timescales calibration assigned to Scrophulariaceae was obtained from TIMETREE [68,69], with the uppermost limit of the time interval set as a minimum hard bound (mean = 44) that includes the entire geological interval (24–64 MYA). The MCMC chain was run in two separate analyses for 10-million posterior iterations sampling every 1000 posterior iterations. The initial 20% iterations of each analysis were discarded as burn-in before combining. The effective sample sizes (ESS) was assessed by using the Tracer v1.6 (http://beast.bio.ed.ac.uk/Tracer) with values greater than 200 considered as indicating optimal convergence and tree likelihood stationarity. A maximum clade credibility (MCC) tree was constructed in TreeAnnotator v1.8.0 [67] depicting the maximum sum of posterior clade probabilities. The MCC tree was visualized in FigTree v1.4.2 [63]. In addition, the R package “bGMYC” [65] was used to conduct the bGMYC analysis, and 10,000,000 MCMC generations, a thinning of 1000 generations, and 20% burn-in. Starting parameters were set according to the default settings. For the bPTP algorithm, 10,000 random post-burn-in trees from the posterior distribution of Bayesian analysis were used to shed light on species boundaries.

### 3.3. Chloroplast Genome Annotation and Comparisons

The complete cp genomes were annotated with the identification of introns and exons using the online annotation tool DOGMA [70] and plann 1.1 [71]. The positions of start and stop codons and boundaries between introns and exons were investigated according to the published cp genome of *S. takesimensis* (KM590983.1). The annotated GenBank files were used to draw the circular chloroplast genome maps using OrganellarGenomeDRAW 1.2 [72]. The online program mVISTA program [73] was employed in the LAGAN mode to detect the variation within the chloroplast genomes. The cp genome of *B. colvilei* GJ2 was used as a reference. The boundaries between IR and SC regions of these species were also compared and analyzed using the self-by-self comparison from the online visualization tool Circoletto [74] and the sequence visualization modules from UGENE version 1.29.0 [75]. We used Mauve version 2.4.0 [76] to compare the genomes, to filter out the IRb across the five *Buddleja* individuals and to identify the single nucleotide polymorphisms (SNPs). Divergent frequencies of SNPs between species were calculated manually.

### 3.4. Morphological Study and Taxonomy Treatment

Measurements and morphological character assessments of *B. sessilifolia* and *B. colvilei* were taken from both herbarium specimens (obtained from JSTOR Global Plants http://plants.jstor.org, Chinese Virtual Herbarium http://www.cvh.ac.cn, Herbarium, Kunming Institute of Botany, CAS and National Herbarium & Plant Laboratories Government of Nepal, for details of specimens see Table S3), and from field observations of living plants. Morphological measurements were made from two populations for each species (Table S2), from at least 30 individuals (or all the individuals in populations with fewer than 30 individuals) in each population. Principal coordinate analysis (PCoA) was carried out using PAST (Paleontological Statistics) software, version 3.04 [77] to study the correlation of measurements between two populations for each of the two species, as well as the relationships among the four populations. Six morphological characters were used in the PCoA, including length of styles, length and width of fruits, length of corolla tube, and hairs on both ovary and capsules (assigned the value 0 or 1 to represent the absence or presence of hairs). A total of 100 measurements from 15–20 individuals in each population were made, plant heights were made from all the individuals (from 20 to 160, see Table S2), 100 measurements of the length of corolla tube of *B. colvilei* were made from the specimens.

The redescription of *B. sessilifolia* included the newly made observations and measurements along with the initial descriptions from the type specimens [10]. The comparison between *B. colvilei* and *B. sessilifolia* was therefore based on morphological measurements, specimen examination, molecular data, and literature review [6,7,10,11,12,13]. The phenology of *B. sessilifolia* was observed over three years from 2015 to 2017 in the Gaoligong Mountain, Yunnan, China.

## 4. Conclusions

The cp genomes from two *B. colvilei* individuals and three *B. sessilifolia* individuals were sequenced, analyzed, and compared. The phylogenetic analyses constructed with the whole cp genomes, the large single-copy regions (LSC), small single-copy regions (SSC), inverted repeat (IR) and the nuclear genes 18S/ITS1/5.8S/ITS2/28S all supported *B. sessilifolia* as a distinct species. The coalescence-based species delimitation methods (bGMYC, bPTP) implemented with the whole chloroplast datasets also supported *B. sessilifolia* as a distinct species. The results suggested that *B. sessilifolia* is early diverging among the Asian *Buddleja*, with a divergence time of about 14.67 Ma (95% HPD: 12.19–17.15 Ma). The complete cp genomes from two populations of *B. colvilei* and three populations of *B. sessilifolia* were found to have total lengths ranging from 154,202 bp to 154,710 bp, with GC contents from 38.07 to 38.11%. Overall gene contents and arrangements were found to be highly conserved in the two species, however numerous consistent differences were found between species, and a total of 474 SNPs were identified across the two species upon which our taxonomic revision was based. In addition, morphological characters also supported the revision for *B. sessilifolia* as a distinct species from *B. colvilei*, and thus the taxonomic status of *B. sessilifolia* was revised based on fixed morphological and molecular differences.

## Figures and Tables

**Table 1:** General features of *Buddleja colvilei* and *B. sessilifolia* chloroplast genomes.

Species		*B. colvilei*		*B. sessilifolia*		
		GJ2	GJ3	GJ9	GJ10	GJ11
Size (bp)	Total CP genome	154,225	154,228	154,202	154,204	154,710
	LSC region	85,200	85,203	85,179	85,181	85,687
	SSC region	17,920	17,919	17,905	17,905	17,905
	IR region	25,553	25,553	25,559	25,559	25,559
GC content (%)	Total CP genome	38.07	38.07	38.10	38.10	38.11
	LSC region	36.19	36.19	36.21	36.21	36.25
	SSC region	32.30	32.30	32.42	32.42	32.39
	IR region	43.23	43.23	43.23	43.23	43.23

**Table 2:** List of genes present in the cp genomes of *Buddleja colvilei* and *B. sessilifolia*.

Category	Function	Group of Genes Gene Names					
RNA Genes	Ribosomal RNA Genes	*rrn4.5* ^#^	*rrn5* ^#^	*rrn16* ^#^	*rrn23* ^#^		
	Transfer RNA genes	*trnH*-GUG	*trnK*-UUU *	*trnQ*-UUG	*trnS*-GCU	*trnG*-UCC *	*trnR*-UCU
		*tmC*-GCA	*trnD*-GUC	*trnY*-GUA	*trnE*-UUC	*trnT*-GGU	*trnS*-UGA
		*trnG*-GCC	*trnfM*-AUG	*trnS*-GGA	*trnT*-UGU	*trnL*-UAA *	*trnF*-GAA
		*trnV*-UAC *	*trnM*-AUG	*trnW*-CCA	*trnP*-UGG	*trnH*-CAU ^#^	*trnL*-UUG ^#^
		*trnV*-GAC ^#^	*trnI*-GAU *^#^	*trnA*-UGC *^#^	*trnR*-AGC ^#^	*trnN*-GUU ^#^	*trnL*-UAG
Protein genes	Subunits of Photosystem I	*psaA*	*psaB*	*psaC*	*psaI*	*psaJ*	
		*ycf3* **	*ycf4*				
	Subunits of Photosystem II	*psbA*	*psbB*	*psbC*	*psbD*	*psbE*	*psbF*
		*psbH*	*psbI*	*psbJ*	*psbK*	*psbL*	*psbM*
		*psbN*	*psbT*	*psbZ*			
	Subunits of cytochrome	*petA*	*petB* *^#^	*petD* *	*petG*	*petL*	*petN*
	Subunits of ATP synthase	*atpA*	*atpB*	*atpE*	*atpF* *	*atpH*	*atpI*
	Large subunit of RuBisCO	*rbcL*					
	Subunits of NADH dehydrogenase	*ndhA* *	*ndhB* *^#^	*ndhC*	*ndhD*	*ndhE*	*ndhF*
		*ndhG*	*ndhH*	*ndhI*	*ndhJ*	*ndhK*	
	ATP-dependent protease subunit P	*clpP* **					
	Chloroplast envelope membrane protein	*cemA*					
	Translation initiation factor	*infA*					
Ribosomal proteins	Small subunit of ribosome (SSU)	*rps2*	*rps3*	*rps4*	*rps7*	*rps8*	*rps11*
		*rps12* **^#^	*rps14*	*rps15*	*rps16* *	*rps18*	*rps19*
Transcription	Large subunit of ribosome (LSU)	*rpl2* *^#^	*rpl14*	*rpl16* *	*rpl20*	*rpl22*	*rpl23* ^#^
		*rpl32*	*rpl33*	*rpl36*			
	DNA-dependent RNA polymerase	*rpoA*	*rpoB*	*rpoC1* *	*rpoC2*		
Other genes	Maturase	*matK*					
	Subunit of acetyl-CoA	*accD*					
	C-type cytochrome synthesis gene	*ccsA*					
	Component of TIC complex	*ycf1* Ψ ^#^					
	Hypothetical proteins	*ycf2* ^#^					
		*ycf15* Ψ ^#^					

*—Gene containing a single intron; **—Gene containing two introns; ^#^—One gene copy in each IR; Ψ—Pseudogene.

**Table 3:** Morphological comparison between *Buddleja colvilei* and *B. sessilifolia.*

Characteristic	*B. sessilifolia*	*B. colvilei*
Plant	Subshrubs, ca. 1 m high	Shrubs or small trees, 2–6 (−11 m) high
Branchlets	Quadrangular, glabrous	Subterete, with scattered stellate and glandular hairs
Petiole	Almost none	4–10 mm long
Calyx	3–5.5 mm long, smooth and glabrous on both sides	6–8 mm long, outside densely stellate-tomentose with glandular hairs and more or less scattered stellate hairs, inside with glandular hairs.
Corolla	White, pink to pink purple, yellow inside, 7.5–14 mm long, glabrous outside, pilose inside above the middle	Purple to wine red, white inside, 23–30 mm long, scattered glandular and stellate hairs on both sides
Indumentum of ovary and capsules	Glabrous	Densely stellate tomentose

## Supplementary Materials

The following are available online. Table S1: Species of the other 79 plant chloroplast genomes utilized in phylogenetic analysis and their corresponding accession nos. in the Genbank database, Table S2: Information on the plants and populations of *Buddleja colvilei*, *B. sessilifolia* and *B. asiatica* used in this study, Table S3: Information on the specimens of *Buddleja colvilei* and *B. sessilifolia*, Figture S1: The inflorescence and leaves of *Buddleja asiatica*.
