# Supplementary material for: Characterization of the Complete Chloroplast Genomes of Buddleja colvilei and B. sessilifolia: Implications for the Taxonomy of Buddleja L
Source: Molecules. 2018 May 23;23(6):1248. doi: 10.3390/molecules23061248 (PMC6100213; doi:10.3390/molecules23061248)
Supplement: Supplementary file 1 [file molecules-23-01248-s001.pdf]

**Table S1.** Species of the other 79 plant chloroplast genomes utilized in phylogenetic analysis and their corresponding accession nos. in the Genbank database

| Species | Length | GC content | Accession NO. |
|---|---|---|---|
| *Nicotiana tomentosiformis* | 155,745 bp | 37.79% | AB240139.1 |
| *Olea europaea* | 155,657 bp | 37.82% | FN997651.1 |
| *Tectona grandis* | 153,943 bp | 37.89% | HF567871.1 |
| *Salvia miltiorrhiza* | 151,332 bp | 38.02% | HF586694.1 |
| *Lindenbergia philippensis* | 155,103 bp | 37.79% | HG530133.1 |
| *Boea hygrometrica* | 153,493 bp | 37.59% | JN107811.1 |
| *Nicotiana undulata* | 155,863 bp | 37.88% | JN563929.1 |
| *Synthetic construct* | 160,743 bp | 38.31% | JN563930.1 |
| *Sesamum indicum* | 153,338 bp | 38.20% | KC569603.1 |
| *Echites umbellatus* | 153,970 bp | 38.02% | KJ953904.1 |
| *Rhabdadenia biflora* | 153,376 bp | 38.05% | KJ953911.1 |
| *Scrophularia takesimensis* | 152,425 bp | 38.05% | KM590983.1 |
| *Lathraea squamaria* | 150,504 bp | 38.13% | KM652488.1 |
| *Premna microphylla* | 155,293 bp | 37.87% | KM981744.1 |
| *Saracha punctata* | 156,925 bp | 37.68% | KP280050.1 |
| *Dunalia obovata* | 156,559 bp | 37.69% | KP280057.1 |
| *Iochroma tingoanum* | 156,823 bp | 37.67% | KP280175.1 |
| *Iochroma nitidum* | 156,574 bp | 37.70% | KP294386.1 |
| *Vassobia dichotoma* | 157,142 bp | 37.72% | KP294521.1 |
| *Iochroma loxense* | 157,002 bp | 37.67% | KP296185.1 |
| *Dunalia brachyacantha* | 156,730 bp | 37.71% | KP308151.1 |
| *Paulownia coreana* | 154,545 bp | 37.99% | KP718622.1 |
| *Paulownia tomentosa* | 154,540 bp | 37.99% | KP718624.1 |
| *Scrophularia buergeriana* | 153,631 bp | 37.99% | KP718626.1 |
| *Scrophularia takesimensis* | 152,436 bp | 38.05% | KP718628.1 |
| *Dunalia solanacea* | 156,869 bp | 37.65% | KP998157.1 |
| *Salvia rosmarinus* | 152,462 bp | 37.99% | KR232566.1 |
| *Perilla frutescens* | 152,598 bp | 37.83% | KT220689.1 |
| *Perilla citriodora* | 152,602 bp | 37.82% | KT220690.1 |
| *Perilla frutescens* | 152,656 bp | 37.81% | KT220691.1 |
| *Perilla setoyensis* | 152,607 bp | 37.82% | KT220692.1 |
| *Abeliophyllum distichum* | 155,982 bp | 37.82% | KT274029.1 |
| *Scrophularia dentata* | 152,553 bp | 37.95% | KT428154.1 |
| *Veronica nakaiana* | 152,319 bp | 37.93% | KT633216.1 |
| *Veronicastrum sibiricum* | 152,930 bp | 38.29% | KT724053.1 |
| *Gynochthodes nanlingensis* | 154,086 bp | 38.52% | KT852576.1 |
| *Lavandula angustifolia* | 153,448 bp | 38.04% | KT948988.1 |
| *Castilleja paramensis* | 152,926 bp | 38.19% | KT959111.1 |
| *Pedicularis ishidoyana* | 152,571 bp | 38.09% | KU170194.1 |
| *Iochroma cyaneum* | 156,863 bp | 37.68% | KU306396.1 |

| | | | |
|---|---|---|---|
| *Acnistus arborescens* | 156,841 bp | 37.68% | KU306735.1 |
| *Eriolarynx fasciculata* | 157,025 bp | 37.69% | KU306938.1 |
| *Iochroma umbellatum* | 156,730 bp | 37.68% | KU310542.1 |
| *Iochroma lehmannii* | 156,835 bp | 37.65% | KU310617.1 |
| *Iochroma lehmannii* | 156,698 bp | 37.71% | KU310654.1 |
| *Iochroma cardenasianum* | 156,510 bp | 37.90% | KU310932.1 |
| *Iochroma australe* | 156,996 bp | 37.67% | KU311000.1 |
| *Iochroma salpoanum* | 156,971 bp | 37.67% | KU315119.1 |
| *Iochroma cyaneum* | 156,791 bp | 37.69% | KU319588.1 |
| *Iochroma ellipticum* | 157,052 bp | 37.69% | KU323367.1 |
| *Acnistus arborescens* | 156,898 bp | 37.68% | KU568472.1 |
| *Erythranthe lutea* | 153,150 bp | 37.72% | KU705476.1 |
| *Stachys chamissonis* | 150,254 bp | 38.53% | KU724138.1 |
| *Stachys coccinea* | 150,275 bp | 38.49% | KU724139.1 |
| *Stachys sylvatica* | 150,167 bp | 38.55% | KU724140.1 |
| *Dracocephalum palmatum* | 150,510 bp | 37.83% | KU958581.1 |
| *Rehmannia chingii* | 154,055 bp | 37.97% | KX426347.1 |
| *Ilex latifolia* | 157,610 bp | 37.63% | KX426465.1 |
| *Ilex szechwanensis* | 157,822 bp | 37.65% | KX426466.1 |
| *Ilex delavayi* | 157,671 bp | 37.65% | KX426470.1 |
| *Rehmannia glutinosa* | 153,622 bp | 37.95% | KX636157.1 |
| *Rehmannia henryi* | 153,890 bp | 37.95% | KX636158.1 |
| *Rehmannia solanifolia* | 153,989 bp | 37.94% | KX636159.1 |
| *Rehmannia piasezkii* | 153,925 bp | 37.94% | KX636160.1 |
| *Rehmannia elata* | 153,772 bp | 37.97% | KX636161.1 |
| *Haberlea rhodopensis* | 153,099 bp | 37.75% | KX657870.1 |
| *Lysionotus pauciflorus* | 153,856 bp | 37.50% | KX752081.1 |
| *Digitalis lanata* | 153,108 bp | 38.56% | KY085895.1 |
| *Aloysia citrodora* | 154,699 bp | 39.19% | KY085903.1 |
| *Hydrangea petiolaris* | 157,434 bp | 37.82% | KY412466.1 |
| *Schizophragma hydrangeoides* | 157,692 bp | 37.83% | KY412467.1 |
| *Chionanthus retusus* | 155,687 bp | 37.76% | KY582962.1 |
| *Salvia japonica* | 153,995 bp | 38.00% | KY646163.1 |
| *Hesperelaea palmeri* | 155,820 bp | 37.81% | LN515489.1 |
| *Chirita brachytricha* | 153,723 bp | 37.51% | MF177037.1 |
| *Chirita eburnea* | 152,963 bp | 37.63% | MF177038.1 |
| *Primulina liboensis* | 152,989 bp | 37.65% | MF177039.1 |
| *Hydrangea luteovenosa* | 157,494 bp | 37.88% | MF370556.1 |
| *Nicotiana attenuata* | 155,886 bp | 37.86% | MF577082.1 |

**Table S2.** Information on the plants and populations of *Buddleja colvilei*, *B. sessilifolia* and *B. asiatica* used in this study

| **Name** | **No.** | **Location** | **Short for Population** | **Altitude** (m. a. s. l.) | **longitude** | **Latitude** | **No. of plants** |
|---|---|---|---|---|---|---|---|
| *Buddleja sessilifolia* | GJ9 | Sandui, Dulongjiang village, Gongshan country, China | SD | 2620 | 98°25′24′′ | 27°42′55′′ | -- |
| | GJ10 | On the border to Myanmar, Danzhu village, Gongshan country, China | DZ | 2850 | 98°35′51′′ | 27°37′56′′ | 160 |
| | GJ11 | Fugong to Myanmar border, Mayanmar | MF | 3060 | 98°39′59′′ | 27°14′28′′ | -- |
| | -- | 43 kilometers from Gongshan to Dulongjiang, Gongshan country, China | K | 3140 | 98°29′52′′ | 27°46′48′′ | 20 |
| *B. colvilei* | GJ2 | Goruwale, Meichi, Nepal | GO | 2770 | 87°55′56′′ | 27°05′58′′ | 57 |
| | GJ3 | Yadong, Tibet, China | YD | 3160 | 88°58'20" | 27°21'29" | 73 |
| *B. asiatica* | GJ1 | Kunming Botanical Garden, Kunming, Yunnan, China | KB | 1910 | 102°44'56" | 25°8'42" | -- |

**Table S3.** Information on the specimens of *Buddleja colvilei* and *B. sessilifolia*

| Species Name | Source | Herbarium | Specimens No. | Resource Type |
|---|---|---|---|---|
| *Buddleja colvilei* | JSTOR Global Plants http://plants.jstor.org (Jstor) | Royal Botanic Gardens, Kew (K) | K001096390 | - |
| | Jstor | K | K001096391 | Paratype |
| | Jstor | Universität Göttingen | GOET005437 | Isosyntype |
| | Jstor | Natural History Museum (BM) | BM000997790 | Type? |
| | Jstor | Muséum National d'Histoire Naturelle | P02428164 | - |
| | Jstor | Herbarium Russian Academy of Sciences - V. L. Komarov Botanical Institute | LE00016140 | Syntype |
| | Jstor | K | K001096392 | Paratype |
| | Jstor | Herbarium of the Arnold Arboretum | A00075849 | Isolectotype |
| | Jstor | Botanische Staatssammlung München | M0183827 | Isosyntype |
| | Jstor | BM | BM000997788 | Isosyntype |
| | Jstor | Swedish Museum of Natural History Department of Botany | S10-26002 | Syntype |
| | Jstor | The Gray Herbarium | GH00075848 | Isolectotype |
| | National Herbarium & Plant Laboratories (NHPL) Government of Nepal | Herbarium Universitatis Tokyoensis | 151396 | - |
| | NHPL | His Majesty's Govt. of Nepal Department of Medicinal Plants, Kathmandu, Nepal Herbarium | 15510 | - |

| | | | | |
|---|---|---|---|---|
| | NHPL | His Majesty's Govt. of Nepal Department of Medicinal Plants, Kathmandu, Nepal Herbarium | 466 | - |
| | NHPL | Herbarium Universitatis Tokyoensis | 6303886 | - |
| | Herbarium, Kunming Institute of Botany, CAS (KUN) | KUN | 9263254 | - |
| *B. sessilifolia* | Chinese Virtual Herbarium http://www.cvh.ac.cn (CVH) | Herbarium, Institute of Botany, CAS (PE) | 323355 | type |
| | CVH | PE | 2456688 | - |
| | CVH | PE | 1283441 | - |
| | CVH | PE | 1283396 | |
| | CVH | PE | 1283006 | |
| | KUN | KUN | 0763814 | |
| | KUN | KUN | 0789569 | |

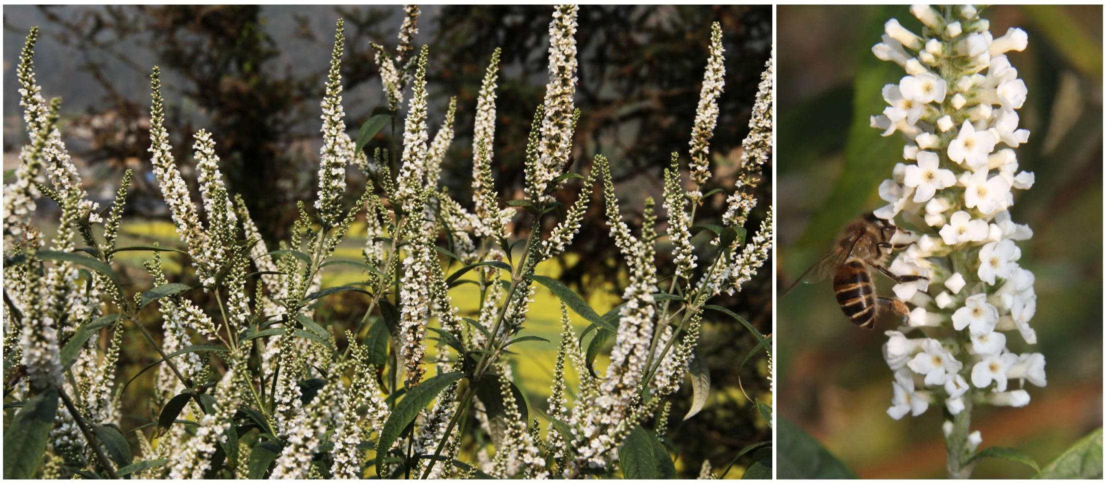

**Figure S1.** The inflorescence and leaves of *Buddleja asiatica*
